# Reducing potentially preventable complications at the multi hospital level

**DOI:** 10.1186/1756-0500-4-271

**Published:** 2011-07-29

**Authors:** Ronald J Lagoe, Gert P Westert, Anne Marie Czyz, Pamela E Johnson

**Affiliations:** 1 Hospital Executive Council, Syracuse, New York, USA; 2 National Institute of Public Health and the Environment, Bilthoven, Netherlands; 3 St. Joseph's Hospital Health Center, Syracuse, New York, USA; 4 Community General Hospital, Syracuse, New York, USA

## Abstract

**Background:**

This study describes the continuation of a program to constrain health care costs by limiting inpatient hospital programs among the hospitals of Syracuse, New York. Through a community demonstration project, it identified components of individual hospital programs for reduction of complications and their impact on the frequency and rates of these outcomes.

**Findings:**

This study involved the implementation of interventions by three hospitals using the Potentially Preventable Complications System developed by 3M™ Health Information Systems. The program is noteworthy because it included competing hospitals in the same community working together to reduce adverse patient outcomes and related costs.

The study data identified statistically significant reductions in the frequency of high and low volume complications during the three year period at two of the hospitals. At both of these hospitals, aggregate complication rates also declined. At these hospitals, the differences between actual complication rates and severity adjusted complication rates were also reduced.

At the third hospital, specific and aggregate complication rates remained the same or increased slightly. Differences between these rates and those of severity adjusted comparison population also remained the same or increased.

**Conclusions:**

Results of the study suggested that, in one community health care system, the progress of reducing complications involved different experiences. At two hospitals with relatively higher rates at the beginning of the study, management by administrative and clinical staff outside quality assurance produced significant reductions in complication rates, while at a hospital with lower rates, management by quality assurance staff had little effect on reducing the rate of PPCs.

## Introduction

In Europe and the United States, continued interest is focusing on reducing adverse health care outcomes as a means of containing costs, as well as improving care. This interest is being stimulated by increasing expenses for government and private sector health care funders [1,2].

In the United States, the economic recession that began in 2008 and the enactment of health care reform legislation have triggered the development of new efforts to contain expenses by improving patient outcomes in this area. In his Inaugural Address, President Barack Obama called for higher quality of health care at reduced costs [3]. Since that time, health care reform legislation enacted in 2010 provided for implementation of Medicare Accountable Care Organizations and other programs which stimulate higher quality of care by contracting for fixed reimbursement rates across communities and networks [4-6].

The shift in power within the United States Congress that occurred in November 2010 will probably not diminish interest in containing health care costs by improving outcomes. Substantial Republican gains in the Congress were produced, in large part, by voter interest in reduced government spending [7].

Although interest in containing health care spending is often focused at national and state levels because of public and private payors, the mechanisms of the process often play out among providers in local communities. Much of it concerns the types of staff and the data mechanisms used to manage outcomes within individual hospitals [8,9].

This study describes the continuation of a program to constrain health care costs by limiting inpatient hospital programs among the hospitals of Syracuse, New York. Through a community demonstration project, it identifies components of individual hospital programs for reduction of complications and their impact on the frequency and rates of these outcomes.

## Methods

This study describes the continuation of a program to improve hospital outcomes in the hospitals of Syracuse, New York using the Potentially Preventable Complications (PPC) System developed by 3M™ Health Information Systems. This area includes a resident population of 446,065 and four hospitals, three of which participated in the study. The program is noteworthy because it involves a group of competing hospitals in the same community working together to reduce adverse patient outcomes and related costs [10].

The initial component of this program involved identification of the increased costs generated by inpatient complications. A major driver of the program in the Syracuse hospitals was the potential for reducing the costs of PPCs. Analyses of patients assigned to the same All Patients Refined Diagnosis Related Groups and severity of illness categories demonstrated that those who experienced PPCs had actual costs three to four times higher than those who did not [10].

The implementation of programs to address inpatient complications in the Syracuse hospitals occurred between October 2008 and June 2009. At each hospital, the program included the following components based on PPCs [10].

PPC Data Development and Distribution

Educational Programs for Staff

Clinical Record Reviews to Verify Complications

Selection of Objectives by PPC Category

Identification of Drivers of Complications

Identification and Implementation of Interventions

Since June 2009, the community wide program to address inpatient complications has evolved and matured. This study describes that process at each of the participating hospitals and suggests relevant conclusions.

### Community General Hospital

Between October 2008 and September 2010, efforts to reduce PPCs at Community General Hospital focused on clostridium difficile colitis, urinary tract infections, post hemorrhage and acute anemia, pulmonary embolism, and venous thrombosis. These efforts were managed by the Chief Fiscal Officer and the Infection Control Manager with support from the Department of Quality Assurance. Throughout the demonstration project, monthly data were provided by the Hospital Executive Council.

Reduction of infections for clostridium difficile colitis has been a focus at the Hospital. Much of this activity has involved environmental efforts to control this organism. Education of the environmental services department staff through the Office of Infection Control has been a key to preventing its spread. In 2009, terminal cleaning times for patient rooms, toilet rooms, showers, medication rooms, kitchens, and utility spaces were updated. In addition, policies and procedures for identification and management of this infection were revised and updated. The Office of Infection Control and the Hospital Epidemiologist have monitored antibiotic use and assign patients with diarrhea to private rooms.

Urinary tract infection has been the PPC with the highest rate per 1,000 discharges at the hospital, although its incidence has declined. In order to address this complication, procedures for management of patients with foley catheters were implemented through the Office of Infection Control in September 2008. Since that time, the Office has aggressively managed implementation of these procedures and reviewed PPCs that were identified for this infection.

Complications related to post hemorrhagic and acute anemia with transfusions were found to be related to a medical staff practice pattern for orthopedic patients discharged to nursing homes during 2009. Since that time, the practice pattern has been revised through cooperation among the Chief Fiscal Officer, the Chief of Orthopedics, and the physicians involved. This occurred through review of transfusion policies and communication of guidelines to surgeons and physician assistants.

Venous Thrombosis and Pulmonary Embolism have been the subject of efforts at Community-General Hospital because of the severity of these complications. Review of PPC data for these diagnoses by the Chief Fiscal Officer and the Office of Infection Control have not identified any patterns by service area. Between 2008 and 2009, efforts to improve care for deep venous thrombosis resulted in a change in vendors for PICC line catheters and the use of more large diameter catheters by the hospital administration and medical staff. Subsequent review of experience resulted in a return to the original vendor and greater use of small bore catheters in 2010. In 2010, risk assessment and prophylaxis order forms for venous thrombosis and pulmonary embolism were revised and implemented throughout the hospital.

### Crouse Hospital

Efforts to reduce PPCs at Crouse Hospital have focused on urinary tract infection, decubitus ulcer, pulmonary embolism, and central venous catheter infections. These efforts have been managed by specific hospital staff and physician champions through the Quality Assurance Department. The hospital used the PPC software to monitor these efforts during 2008 and 2009, discontinued this process in the initial quarters of 2010, then returned to it in July of that year. For the 2008 - 2009 and the third quarter of 2010, monthly PPC data were provided by the Hospital Executive Council.

PPCs involving urinary tract infection experienced the highest frequency at Crouse Hospital during the period of the study. Interventions that were initiated in 2008 included the placement of stickers in patient charts by the nursing staff which requested the physician to remove the sticker if the patient's condition did not warrant it, or to renew the order if it was. Beginning in 2008, educational programs were implemented by hospital infection control nurses to encourage early removal of urinary catheters. Followup education was provided by these nurses when urinary tract infections were identified. In addition, education was provided to Clinical Documentation Specialists and physicians on the nursing units to ensure that these complications were documented correctly.

PPCs involving decubitus ulcers at Crouse Hospital, although not involving large numbers of patients, have generated long inpatient stays and been addressed by the program. Beginning in 2008, the Inservice Education Department has provided extensive training to physicians and nurses in comprehensive skin assessment, staging of ulcers present on admission, and identification of patients at risk of this complication. Each patient admitted to a critical care or a medical surgical unit received an assessment within 24 hours of admission and daily reassessments. Interventions that were implemented to prevent this complication included specialty mattresses, turning and positioning procedures, nutrition involvement, skin cleansing, moisture management, and use of chair cushions.

Pulmonary Embolism as a PPC has been addressed by a series of interventions at Crouse Hospital beginning in June 2009. This program has been developed and implemented by a physician champion and a hospital pharmacist. A protocol and related form that include a risk assessment was developed for use with each medical/surgical inpatient. The form included recommendations for mechanical and pharmaceutical interventions for each patient. Copies were placed in each patient chart. Interventions included ambulation for patients identified as being at low risk and mechanical and pharmaceutical interventions for patients at moderate to high risk.

PPCs for infections due to central venous catheters have been addressed by a series of initiatives at Crouse Hospital beginning in 2010. These efforts have been managed by the hospital Physician Epidemiologist and two Nurse Administrators with interests in this area. During 2009, a central line bundle was implemented at the hospital, however, infection rates did not improve as anticipated. During 2010, a pilot program was implemented on a single nursing unit including the use of a needle less connector and a PICC line team assuming responsibility for dressing changes, assessment, and connector changes for all central lines.

### St. Joseph's Hospital Health Center

Reduction of PPCs at St. Joseph's Hospital Health Center focused on pneumonia, decubitus ulcer, and post hemorrhagic and other acute anemia with transfusion. The management of this process involved from an administrative vice president, to the nurse managers of inpatient units, to the infection control manger, the respiratory therapy manager, and the product line manager for orthopedics. Beginning in October 2008, this process was supported by monthly reports including PPC data developed by the Hospital Executive Council.

Reduction of inpatient complications for pneumonia was the largest quality assurance challenge at St. Joseph's Hospital Health Center. During 2008, this diagnosis accounted for 18.2 percent of all PPCs at the Hospital.

Between October and December 2008, efforts in this area focused on ventilator associated pneumonia through the implementation of a protocol for management of ventilator patients under the leadership of the Vice Presidents for Nursing and Utilization Management. By February 2009, the incidence of this complication had been effectively eliminated.

During 2009, efforts to reduce the incidence of remaining complications involving hospital acquired pneumonia not involving ventilators were directed by Nurse Managers and other administrative staff. They focused on implementation of interventions such as early ambulation and respiratory care for patients experiencing cardiac surgery and intensive care.

Beginning in October 2009, the Chief Nursing Officer and the Manager of Infection Control became involved with this process. This resulted in organization of a program to address pneumonia as a PPC including the following specific interventions.

- Elevation of head of bed 30 - 45 degrees if not medically contraindicated

- Hourly use of incentive spirometry

- Assistance with ambulation

- Encouragement of cough, deep breathing

- Use of hand hygiene protocol

This program was initially implemented in the first quarter of 2010 through the Office of Infection Control and the Inservice Education Department. Analysis of PPC data for this period demonstrated that many patients in the target population did not receive the interventions. This resulted in a change in management to the Office of Infection Control and the Respiratory Therapy Department. Under this structure, the number of patients receiving the intervention increased substantially. This process was supported through the provision of aggregate and patient specific data for patients with the pneumonia PPC at two week intervals by the Hospital Executive Council.

Reduction of PPCs at St. Joseph's Hospital Health Center also involved decubitus ulcer. This was diagnosis with relatively small volume that added large numbers of days to inpatient stays. The program was managed by the Vice Presidents for Nursing and Utilization. Interventions included the implementation of comprehensive skin assessments for all medical/surgical inpatients on admission. In addition, specialty pressure mattresses were installed in all inpatient beds.

Initial efforts to reduce PPCs at St. Joseph's Hospital Health Center also included post hemorrhagic and other acute anemia with transfusion. This program was managed by the Vice President for Utilization and product line managers for orthopedics and other services. The cause of this complication was determined to be a practice pattern that resulted in the ordering or transfusions for patients requiring long term care immediately prior to discharge. This issue was addressed through revision of guidelines for the patients at risk and through revision of administrative procedures for authorization of transfusions.

Further efforts to manage inpatient complications at St. Joseph's Hospital Health Center will focus on additional complications and interventions. Because of the large size of the adult medical/surgical inpatient population at this hospital, they will also involve early identification of high risk patients to make best use of available resources.

### Study Analysis

The study compared the frequencies and rates of PPCs for each category in which an intervention was implemented in the three Syracuse hospitals. In addition, the frequencies and rates of all PPCs were compared hospital wide. The comparisons involved January - September 2008 - 2010. The 2008 period included the nine months immediately prior to the implementation of interventions, while the 2009 and 2010 periods included their impact.

In order to evaluate the impact of changes in the composition of the hospital populations for inpatient complications, the analysis also included comparisons of hospital complication rates with those of benchmark populations with the same severity of illness. The benchmark populations were based on California and New York State inpatient data. In the analyses p = <0.05 was the accepted alpha level employed in the methodology.

## Results

Data for both of the study analyses are summarized in Tables 1 and 2.

**Table 1:** Potentially Preventable Complications Select Categories Syracuse Hospitals January - September 2008 - 2010

	Patients with PPC			PPC Rate Per 1,000 Discharges			Percent Difference	p value
	2008	2009	2010	2008	2009	2010	2008 - 2010	
Community General								
Hospital								
Clostridium Difficile Colitis	16	16	9	2.66	2.86	1.73	-34.96	0.05
Urinary Tract Infection	68	48	31	12.47	9.45	6.37	-48.92	0.04
Post-Hemorrhage & Other Acute Anemia	27	37	0	5.61	7.11	0	-100	0.01
Venous Thrombosis	13	11	10	2.17	0.54	1.94	-10.6	0.11
Pulmonary Embolism	5	4	7	0.84	0.72	1.36	61.9	0.05
								
Crouse Hospital								
Urinary Tract Infection	76	99	79	6.5	9.4	6.59	1.38	0.15
Decubitus Ulcer	9	11	10	0.72	0.99	0.78	8.33	0.25
Pulmonary Embolism	17	10	20	1.45	0.94	1.56	7.59	0.22
Infection Due to Central Venous Catheters	9	11	10	0.69	0.94	0.75	8.7	0.31
								
St. Joseph's Hospital								
Health Center								
Pneumonia & Other Lung Infections	149	125	125	13.69	12.69	11.06	-19.21	0.05
Post-Hemorrhage & Other Acute Anemia	86	22	0	7.82	2.15	0	-100	0.01
Decubitus Ulcer	45	20	35	3.35	1.64	2.48	-25.97	0.05
								
Total with One or More PPCs								
Community General Hospital	295	253	172	48.73	44.99	32.36	-33.59	0.04
Crouse Hospital	416	491	503	31.17	41.52	35.96	15.37	0.07
St. Joseph's Hospital Health Center	820	702	778	58.17	53.99	48.15	-17.23	0.05

Source: Hospital Executive Council data.

**Table 2:** Potentially Preventable Complication Rates Select Categories Syracuse Hospitals January - September 2008 - 2010

	PPC Rate Per 1,000 Dchgs Percent Difference	p Value	PPC Rate Difference from Severity Adjusted Comparison Benchmark			Rate Difference
	2008 - 2010		2008	2009	2010	2008 - 2010
Community General						
Hospital						
Clostridium Difficile Colitis	-34.96	0.05	1.42	1.62	0.49	-0.93
Urinary Tract Infection	-48.92	0.04	1.19	-1.72	-4.99	-6.18
Post-Hemorrhage & Other Acute Anemia	-100	0.01	2.28	3.71	-3.25	-5.53
Venous Thrombosis	-10.6	0.11	0.34	0.14	0.04	-0.3
Pulmonary Embolism	61.9	0.05	-0.1	-0.22	0.38	0.48
Crouse Hospital						
Urinary Tract Infection	1.38	0.15	-0.54	2.11	1.09	1.63
Decubitus Ulcer	8.33	0.25	-0.12	0.17	-0.11	0.01
Pulmonary Embolism	7.59	0.22	0.8	0.26	0.86	0.06
Infection Due to Central Venous Catheters	8.7	0.31	0.69	0.75	0.75	0.06
St. Joseph's Hospital Health Center						
Health Center						
Pneumonia & Other Lung Infections	-19.21	0.05	4.96	3.38	1.73	-3.23
Post-Hemorrhage & Other Acute Anemia	-100	0.01	5.73	-0.13	-2.3	-8.03
Decubitus Ulcer	-25.97	0.05	2.01	0.57	1.03	-0.98
Total with One or More PPCs						
Community General Hospital	-33.59	0.04	7.93	4.03	4.57	-3.36
Crouse Hospital	15.37	0.07	-6.82	3.35	3.1	9.92
St. Joseph's Hospital Health Center	-17.23	0.05	7.45	-0.59	1.31	-6.14

Benchmark data based on California and New York State inpatient hospital discharges, 2007.

Source: Hospital Executive Council data.

The data in Table 1 demonstrated that the impact of the interventions varied widely among the three hospitals. Statistically significant reductions occurred in the frequency of high volume complications such as urinary tract infection at Community-General Hospital and pneumonia at St. Joseph's Hospital Health Center. Non significant changes in the rate of urinary tract infection were identified at Crouse Hospital.

Statistically significant reductions in low frequency complications such as clostridium difficile colitis and pulmonary embolism occurred at Community-General Hospital and decubitus ulcer at St. Joseph's Hospital Health Center while non significant changes were identified in low frequency complications were identified at Crouse Hospital. At the same time, the differences between hospital complication rates for decubitus ulcer at Crouse Hospital were lower than those at St. Joseph's Hospital Health Center.

The data in Table 2 demonstrated that these changes were reflected in the differences between hospital rates and severity adjusted comparison populations. The differences declined at Community General and St. Joseph's Hospitals, while remaining unchanged or increasing slightly at Crouse Hospital.

At the aggregate level, the data in Table 1 identified statistically significant declines at Community General and St. Joseph's Hospitals and a non significant increase at Crouse Hospital. The aggregate complication rates at Crouse Hospital were lower than those at the other hospitals between 2008 and 2009, while the complication rate at Community-General Hospital was the lowest identified during 2010.

The data in Table 2 demonstrated that differences in aggregate hospital complication rates and those of severity adjusted comparison populations declined from 2008 to 2010 at Community-General Hospital and St. Joseph's Hospital Health Center. The rates at Crouse Hospital increased between 2008 and 2009, then declined between 2009 and 2010.

## Discussion

This study evaluated the experience of three competing hospitals in a single United States metropolitan area reducing PPCs. The study focused on the impact of the evolution of a series of interventions on the frequencies and rates of high and low volume complications that were addressed by interventions at the hospitals over an extended (three year) period of time. Because the hospital interventions addressed different groups of complications, general conclusions were identified.

Although the study involved the use of a standardized evaluation algorithm for complications that was applied to hospitals in the same metropolitan area, a number of limitations related to its conclusions. As noted in the Methods, the hospitals employed different interventions to achieve reductions in complications. These interventions were based on conclusions regarding drivers of complications identified in the PPC data. Different education programs were employed to address specific complications based on the needs of staff involved in each initiative. Additionally, the management structures of the hospitals were not identical. The degree of staff engagement with respect to the project varied from hospital to hospital based on the commitment of each institution to the project.

Results of the study identified statistically significant reductions in hospital stays for both high and low volume PPCs. Significant reductions in specific and overall complication rates were identified at Community-General Hospital and St. Joseph's Hospital Health Center. During the period of the study, differences between hospital inpatient complications rates and those of severity adjusted comparison populations declined then increased slightly at both of these hospitals, resulting in overall declines for the three year period. A review of the development of hospital programs between 2008 and 2010 suggested that reductions in complication rates occurred where the processes were managed by administrative staff such as a Chief Fiscal Officer or Vice President for Clinical Services and the Offices of Infection Control.

Practice changes eliminated the incidence of post hemorrhagic and acute anemia with transfusion at two of the hospitals. Unlike the PPCs resulting from infections, this complication was amenable to such interventions.

Results of the study also indicated that Crouse Hospital, which did not experience significant reductions in complications rates, produced rates below those of the other hospitals for one high volume and one low volume complication that were addressed by interventions and at the aggregate level for PPCs. Differences between hospital complication rates and those of severity adjusted comparison increased slightly for most PPCs and at the aggregate level. During this period, the management of inpatient complications at Crouse Hospital was carried out by the quality assurance staff.

These results suggest that, in one community health care system, the progress of reducing complications involved different experiences. At two hospitals with relatively higher rates at the beginning of the study, management by administrative and clinical staff outside quality assurance produced significant reductions in complication rates, while at a hospital with lower rates, management by quality assurance staff had little effect on reducing the rate of PPCs. These trends were supported by the declines in differences between hospital and severity adjusted comparison population rates at two of the Syracuse hospitals and non significant increases at a third.

These results suggest that different types of inpatient outcomes may require different types of management staff. Further community based research will be necessary to identify the best management approaches to reducing inpatient complications. This research will also need to address additional complications and interventions, as well as early identification of high risk patients. The PPC software provides a standardized tool for evaluation of the development of these outcomes within and among hospitals.

## Competing interests

The authors declare that they have no competing interests.

## Authors' contributions

RJL was responsible for the study design, data analysis, and development of the manuscript. GPW contributed to the study design and development of the manuscript. AMC and PJ were responsible for implementation of the project at individual hospitals. All of the authors have read and approved the final manuscript.
